# Attention Deficit/Hyperactivity Disorder and Urinary Nonylphenol Levels: A Case-Control Study in Taiwanese Children

**DOI:** 10.1371/journal.pone.0149558

**Published:** 2016-02-18

**Authors:** Ching-Jung Yu, Jung-Chieh Du, Hsien-Chih Chiou, Shang-Han Yang, Kai-Wei Liao, Winnie Yang, Ming-Yi Chung, Ling-Chu Chien, Betau Hwang, Mei-Lien Chen

**Affiliations:** 1 Institute of Environmental and Occupational Health Sciences, School of Medicine, National Yang Ming University, Taipei, Taiwan; 2 Department of Pediatrics, Taipei City Hospital, Zhongxiao branch, Taipei, Taiwan; 3 Department of Child and Adolescent Psychiatry, Taipei City Hospital, Songde branch, Taipei, Taiwan; 4 Department of Pediatrics, Taipei City Hospital, Yangming branch, Taipei, Taiwan; 5 Department of Life Sciences and Institute of Genome Sciences, National Yang Ming University, Taipei, Taiwan; 6 School of Public Health, Taipei Medical University, Taipei, Taiwan; Hospital Universitario LA FE, SPAIN

## Abstract

**Objective:**

Nonylphenol (NP) belongs to the family of endocrine disruptors, and it is widely used in industrial applications and is ubiquitous in daily foods. Animal studies have suggested that NP exposure might promote motor hyperactivity, likely by causing deficits in dopaminergic neurons. However, research assessing NP exposure and epidemiology studies on human populations are limited. The aim of this study was to explore the association between child NP exposure and ADHD while considering particular covariants, such as lead levels and dopamine-related gene variations.

**Methods:**

A case-control study was conducted on patients with clinically diagnosed ADHD; the Swanson, Nolan and Pelham, Fourth Revision (SNAP-IV) questionnaire was used to identify normal controls aged 4–15 years. Participants were examined for urinary NP concentrations, blood lead levels, and select single-nucleotide polymorphisms of two dopamine-related genes (D4 dopamine receptor, *DRD4*, and dopamine transporter, *DAT1*). Socio-demographic variables, maternal lifestyle factors during pregnancy and family medical history were obtained using a questionnaire.

**Results:**

A total of 97 children with doctor-diagnosed ADHD and 110 normal controls were enrolled. The blood lead levels in both groups were similar (1.57±0.73 vs. 1.73±0.77 μg/dL, *p* = 0.15). No significant difference in urinary NP concentration was found between the children with ADHD and the control subjects (4.52±3.22 μg/g cr. vs. 4.64±2.95 μg/g cr., *p* = 0.43). ADHD was significantly more prevalent among males in this study (male to female ratio: 5:1 for the ADHD group and 1.3:1 for the control group, *p*<0.01). The analysis was repeated after excluding the females, but this had no effect on the association between NP and ADHD. The regression model, including or excluding females, indicated no increased odds of having ADHD in the context of NP exposure after adjusting for covariants.

**Conclusion:**

This study indicated that NP exposure might not promote ADHD in children, even though children in Taiwan had relatively high levels of NP compared to those reported previously and those in developed nations.

## Introduction

Nonylphenol (NP) and its parent compound, nonylphenol polyethoxylate (NPnEO), are used in various manufacturing processes and in a wide range of products to which humans are exposed [1–5]. The annual global production of alkylphenol polyethoxylate (APnEO) is approximately 650,000 tons, and NPnEO represents approximately 80% of this amount [6, 7]. NP is prevalent in the environment and is a known endocrine-disrupting chemical with estrogen-like effects [8–10]. Estrogen participates in various steps of cellular differentiation and affects the maturation of dopamine neurons [11]. After exposure, NP binds to estrogen receptors and can influence estrogen functions in the developmental stages of animals and humans [5, 12–15].

Humans may be exposed to NP through drinking water; the intake of contaminated foods; the inhalation of air; and absorption via the skin [6, 16]. NP is widespread in Taiwanese food, and the average daily intake of NP is 4- to 8.5-fold higher than that in other countries [17]. In addition, biological monitoring has revealed significant levels of NPs in the adipose tissue, plasma and urine of neonates, children, pubertal students, pregnant mothers, women, and workers in the textile and housekeeping industries [13, 14, 18–21]. However, a limited number of NP exposure assessments and epidemiology studies on human populations have been conducted [22]. Lopez-Espinosa et al. determined the NP concentrations in 20 non-occupationally exposed women in Spain; 100% of these women had detectable levels of NP. Body mass index (BMI) was associated with NP concentration [21]. NP exposure was also found to be associated with an increased risk of low neonatal weight and was shown to disturb pubertal development in Taiwan [13, 14]. Infants and children in Taiwan are constantly exposed to NP, and the health implications of NP exposure for vulnerable children should be monitored.

Attention deficit hyperactivity disorder (ADHD) is one of the most common childhood neurobehavioral conditions, with a prevalence ranging from 5.9%-7.1% worldwide and from 7.5%-9.9% in Taiwan [23, 24]. ADHD interferes with learning and social development; this condition typically begins during the preschool years and often persists into adulthood. The etiology of ADHD remains unclear; however, it is believed to involve individual genetic and environmental factors as well as interactions between these factors [25–27]. The heritability of ADHD has been estimated to be as high as 76% [28], and various polymorphisms in dopamine-related genes have been found to increase the risk of ADHD [29, 30]. The associations between polymorphisms in the dopamine 4 receptor (*DRD4*) and dopamine transporter (*DAT1*) genes and ADHD have been most frequently verified [31]. Lead is one of the most well-studied environmental pollutants [32]. Low-level lead exposure has been associated with a clinical diagnosis of ADHD in several recent studies [31, 33–35].

Animal studies have revealed that the chronic application of NP sensitizes mouse brains and causes neuronal apoptosis [36]. Intracisternal NP injection might promote motor hyperactivity, likely by causing deficits in dopaminergic neurons [37, 38]. These animal investigations suggested that NP exposure was associated with adverse neurodevelopmental outcomes, such as hyperactivity. Several studies have focused on the health implications of ADHD and exposure to potential environmental pollutants, such as lead, phthalates and pesticides, in young and school-age children [35, 39–41]. To the best of our knowledge, no studies have assessed the possible risks of ADHD associated with childhood exposure to NP. The purpose of this study was to explore the associations between NP exposure and ADHD by measuring the urinary concentrations of NP in both ADHD and non-ADHD children. We also considered known potential variables in the etiology of ADHD, such as blood lead levels (BLLs), genetic variations, parental psychosocial factors, family history of nervous system diseases and prenatal exposure to tobacco and alcohol.

## Materials and Methods

### Study subjects

The study protocol was approved by the Taipei City Hospital institutional review board. All participants gave verbal or written assent. Written informed consent was obtained from the participant’s parents or guardians. We recruited subjects between 4 and 15 years of age in outpatient waiting rooms at Taipei City Hospital. The case subjects in this study were children with ADHD identified by board-certified pediatricians or psychiatrists after at least a three-visit clinical assessment. Consecutive children admitted for initial or follow-up ADHD treatment were recruited as cases during the study period, with the exclusion criteria of neurological deficits or mental retardation. The diagnosis of ADHD was performed in accordance with the criteria of the Diagnostic and Statistical Manual of Mental Disorders, 4th edition, revised criteria (DSM-IV-TR) [42].

Control subjects were recruited by randomly selecting healthy children without ADHD aged 4–15 years who utilized Taipei City Hospital for non-ADHD-related visits during the same study period. The same exclusion criteria for the cases were applied to the controls. Controls were screened for the absence of ADHD symptoms based on assessments of their behavior at home and in the classroom by the parent(s) and teachers, respectively, according to the Chinese version of the Swanson, Nolan and Pelham, Fourth Revision (SNAP-IV) questionnaire [43–46]. The rating results were computed by pediatricians and examined for the absence of ADHD symptoms. The SNAP-IV Parent and Teacher Form uses the same format and directly adopts the DSM-IV symptoms; this form has been translated into Chinese and has been found to be a reliable and valid tool for screening for ADHD for clinical and research purposes in Taiwan [45, 46]. The 26-item SNAP-IV is based on a four-point (0–3) scale. It consists of the DSM-IV criteria for inattention (items 1–9), hyperactivity/impulsivity (items 10–18) as the symptoms for ADHD, and oppositional symptoms (items 19–26) characteristic of oppositional defiant disorder. If at least six of the nine inattention items or hyperactivity/impulsivity items were scored at 2 (quite a bit) or 3 (very much) in either the parent or teacher form, the children were defined as having the possible presence of ADHD syndromes [47]. Children scored as having possible ADHD were referred to pediatricians or psychiatrists for further assessment. All the cases were also assessed by SNAP-IV scoring at the initial visit.

Ninety-seven ADHD subjects (N = 97) and one hundred ten normal controls (N = 110) were recruited. The response rates were 54.8% (97/177) and 52.6% (110/209) in the case and control groups, respectively. In this case-control study, the participating subjects also were investigated by questionnaires regarding demographic features, lifestyle factors of the mother during pregnancy and family history of nervous system diseases. Subjects provided a spot urine sample and blood (or saliva) sample during the clinic visit.

### Sample preparation and analysis

#### Reagents

4- Nonylphenol (p-isomers >85%) was obtained from Fluka (Buchs, Switzerland). Methanol, n-hexane, acetone, acetonitrile, hydrochloric acid, acetic acid, and ammonium acetate were LC grade, and all were obtained from Merck (Darmstadt, Germany). β-Glucuronidase (type H-2) was purchased from Sigma-Aldrich (USA). Deionized water, obtained using a Millipore water purification system (Bedford, MA, USA), was prepared before use and was collected in a glass container.

#### Instrumentation

High-performance liquid chromatograph (HPLC) coupled with a fluorescence detector (Hitachi, Tokyo) was used for NP analysis. The reverse-phase column was a Luna C18 (250 x 4.6 mm) with a 5-μm particle size. The isocratic mobile phase was a mixture of acetonitrile and water (75:25, v/v) with a 1.0 mL/min flow rate. The fluorescence detector was operated at an excitation wavelength of 275 nm and an emission wavelength of 300 nm. The samples were injected at volumes of 20 μL.

#### Sample pretreatment and analysis

This study adopted the same NP analytical method used previously in this lab [20]. Before testing, the urine samples were thawed and homogenized using a vortex mixer. The pH values of all the urine samples were adjusted to 5.5 with 1 M acetic acid, and the samples were mixed with 1 mL of 1 M ammonium acetate solution. The urine samples were deconjugated by adding 125 μL of β-glucuronidase to detect total NP (free and conjugated forms). It was followed by incubation at 37°C for 15 h in a shaker bath. The mixture was acidified to pH 3.0 using a 1 M HCl solution. Samples were cleaned using pH solid-phase extraction (SPE) cartridges (3 mL, RP, Supelco, USA), which had been preconditioned with 20 mL of methanol, followed by 3 mL of pure water that was acidified with 1 M HCl solution to pH 3.0. After sample application, each cartridge was washed with 5 mL of pure water. The absorbed compounds on the cartridge were eluted with 3 mL of methanol and were evaluated by HPLC. The NP recovery rate was in the range of 81%-107%, with a CV (coefficient of variance) of 6.7%. The LOD (limit of detection) for NP was 1.6 μg/L.

To avoid contamination of NP, glassware was used for the urine collection for both the case and control groups. Urine samples were sealed, chilled, and transported to the lab immediately. No NPnEO (which is biodegraded to NP)-containing detergents and plastics were used during sample collection, preparation and analysis. NP concentrations were adjusted using creatinine concentrations to correct for variable urine dilutions in the spot urine samples. Urinary creatinine concentrations were determined using a commercially available diagnostic enzyme method (Eagle Diagnostic, USA) that is based on a modified Jaffe reaction [48]. The concentrations of urinary NP were expressed as μg per gram creatinine (μg/g cr.). We excluded one child, an ADHD case, with extremely dilute urine (creatinine levels <30 mg/dL).

Peripheral blood was drawn using a syringe or venoclysis needle, which was then sealed in a heparin-containing vacuum tube and immediately transported at 4°C to the laboratory. One milliliter of whole blood was frozen separately and stored for lead analysis. The BLLs were measured using inductively coupled plasma-mass spectrometry (Thermo, USA). Trace Elements Serum L-2 (Seronorm^TM^, Norway) was used to verify the precision and accuracy of the analytical measurements. For the BLL measurements, the LOD was 0.001 μg/dL with a CV of 3.2%.

The following quality controls were adopted for the NP and BLL measurements: a minimum of five calibration concentrations were used for each batch with correlation coefficients (R^2^) greater than 0.995, and each specimen was calibrated and adjusted using a blank sample. The results are reported as the mean of two replicate measurements. Moreover, quality control samples were analyzed in each batch.

In the situation in which individuals were not able to supply a blood sample, DNA was extracted from a saliva sample. Saliva was spat into the Saliva DNA Collection and Preservation Kit (Norgen Biotek Corporation, Canada) and stored at room temperature until analysis.

#### Gene polymorphisms

Genotype analyses with haplotype-tagging SNPs of *DRD4* and *DAT1* were selected by screening the CHB (Han Chinese in Beijing) panel from the HapMap database (http://hapmap.ncbi.nlm.nih.gov/). To avoid redundancy and to obtain complete genetic coverage, we evaluated linkage disequilibrium patterns and set a maximum r^2^ threshold of 0.8 for all SNPs that possessed a minor allele frequency (MAF) ≥0.05. The non-synonymous SNPs were further inspected using the University of California at Santa Cruz (UCSC) genome browser (http://genome.ucsc.edu/). For the tagged SNPs, MAF values ≥0.1 were used to check the genetic variations in the CHB by browsing the 1000 GENOME database (http://www.1000genomes.org/).

Genomic DNA from peripheral blood leukocytes was extracted by using a DNA mini kit (Geneaid, CA, USA). DNA from saliva was isolated using a QIAamp DNA Mini Kit (Qiagen, Hilden, Germany) according to the manufacturer’s instructions. Genotyping of the *DRD4* and *DAT1* SNPs was performed using the MassARRAY Platform (Sequenom, CA, USA) at the VYM Genome Research Center, National Yang-Ming University. Amplifications were performed in a 384-well polymerase chain reaction system at a minimum concentration of 10 ng/μL DNA. The genotypes were tested for deviation from Hardy-Weinberg equilibrium, and associations were analyzed using standard chi-square goodness-of-fit tests (http://ihg.gsf.de/cgi-bin/hw/hwa1.pl).

#### Covariates

We examined covariates and potential confounders for the association between NP exposure and ADHD. Predictors were chosen based on their association with ADHD in previous studies. The following variables were considered as potential confounders: BLLs, dopamine-related gene variations, age, gender, BMI, maternal age at childbirth, gestational age at birth (<37 weeks or ≥37 weeks), parental education (high school education and below or college or advanced training), maternal smoking during pregnancy (yes or no) and maternal drinking during pregnancy (yes or no) [15, 39, 49–53]. In addition to environmental risk factors, we also included family history of nervous system disease as a covariate. The family history of nervous system diseases listed in the questionnaire includes Parkinson's disease, Alzheimer's disease, ADHD, mental retardation, cerebral palsy, autism, epilepsy, developmental delay, multiple sclerosis and peripheral neuromuscular disease in grandparents, parents or siblings of the subject. BLLs and genetic variations in *DRD4* and *DAT1* were measured in this study, and the other variables were obtained from clinical records or questionnaires completed by the parents.

#### Statistical analysis

SPSS version 17.0 was used for the statistical analysis. Measurements below the LOD were given a value corresponding to LOD/2. We assessed the significance of differences between the case and control groups using the 2-sided nonparametric statistical Mann-Whitney U test for consecutive data and chi-squared tests or Fisher’s exact test for categorical data, where appropriate [54]. The statistical significance was set at *p*<0.05. Logistic regression analyses of NP concentrations and ADHD with and without adjusting for covariant factors were conducted to investigate the odds ratio. Covariates were included in the multivariate analyses if they were related to ADHD at *p*<0.1. A covariate was controlled if the adjusted and unadjusted coefficients between NP exposure and ADHD differed by 10% [55]. Dose-response relationships were analyzed by multivariate logistic regression analyses after grouping the NP concentrations into the <25^th^, 25-75^th^ and >75^th^ percentiles.

## Results

### Demographic characteristics of subjects

Table 1 lists the demographic characteristics of the 207 participating children. Of these participants, 97 were diagnosed with ADHD (81 boys [83.5%] and 16 girls [16.5%]; M:F = 5:1), and 110 were control subjects (63 boys [57.3%] and 47 girls [42.7%]; M:F = 1.3:1). ADHD was significantly more likely to be diagnosed in boys than in girls (χ^2^ = 16.7, *p*<0.01). The mean ages (±SD) of cases and controls were 8.9±2.8 years and 8.9±2.0 years, respectively. Children with and without ADHD did not differ in terms of age, BMI, maternal age at birth, and maternal smoking during pregnancy. Age-related differences were examined, and no differences in the effect estimates were observed.

**Table 1:** Demographic and exposure characteristics of the study participants (N = 207).

Variables		Controls	ADHD	*p*-value
		N = 110	N = 97	
**Gender (%)**				<0.01*
	Male	63 (57.3%)	81 (83.5%)	
	Female	47 (42.7%)	16 (16.5%)	
**Age (years)**		8.9±2.0	8.9±2.8	0.86
**BMI (kg/m**^**2**^**)**		17.3±3.5	17.9±3.6	0.31
**Maternal age at childbirth**		30.3±4.2	30.4±5.5	0.71
**Gestational age at birth (%)**				0.11
	<37 weeks	11 (10.0%)	17 (17.5%)	
	≥37 weeks	99 (90.0%)	80 (82.5%)	
**Paternal education levels (%)**				0.01*
	High School and below	26 (23.6%)	44 (45.4%)	
	College or advanced training	84 (76.4%)	53 (54.6%)	
**Maternal education levels (%)**				0.01*
	High School and below	29 (26.9%)	42 (43.3%)	
	College or advanced training	81 (73.1%)	55 (56.7%)	
**Maternal smoking during pregnancy (%)**				0.16
	No	106 (96.4%)	89 (91.8%)	
	Yes	4 (3.6%)	8 (8.2%)	
**Maternal drinking during pregnancy (%)**				<0.01*
	No	106 (96.4%)	81 (83.5%)	
	Yes	4 (3.6%)	16 (16.5%)	
**Family history of nervous system diseases (%)**				<0.01*
	No	91 (82.7%)	64 (66.0%)	
	Yes	19 (17.3%)	33 (34.0%)	

* *p<*0.05

Control children had significantly higher paternal and maternal education levels compared to children with ADHD. This study also found an association between maternal drinking during pregnancy and ADHD. ADHD cases were significantly associated with a family history of nervous system diseases compared with the reference subjects. Among those variables with *p*<0.1, only maternal education levels and maternal drinking during pregnancy had a greater than 10% coefficient difference in the unadjusted and adjusted regression analyses, and these two variables were adjusted in the logistic regression model.

### Genetic variation influences

Table 2 lists the 10 SNPs that were related to genetic variations in *DRD4* and *DAT1* among the study participants. We observed a nominal significant difference in a *DRD4* gene polymorphism (rs752306) between the case and control groups. However, the difference may have resulted from the limited number of subjects. Logistic regression models of NP and ADHD were estimated after including dopaminergic gene variations (those with *p*<0.1) to determine whether genetic variation influences the risk of having ADHD. Variations in dopaminergic genes did not modify the association between NP exposure and ADHD in this study.

**Table 2:** Polymorphisms in dopamine-related genes (*DRD4/DAT1*) in the study participants (N = 207).

Gene	SNP	Genotypes						
		Control N (%) (N = 110)			Case N (%) (N = 97)			*p*-value
		11	12	22	11	12	22	
*DRD4*	rs7395429	53 (48.2%)	46 (41.8%)	11 (10.0%)	60 (61.9%)	30 (30.9%)	7 (7.2%)	0.07**
	rs3758653	44 (40.0%)	55 (50.0%)	11 (10.0%)	48 (49.5%)	45 (46.4%)	4 (4.1%)	0.09**
	rs11246228	34 (30.9%)	51 (46.4%)	25 (22.7%)	18 (18.6%)	50 (51.5%)	29 (29.9%)	0.05**
	rs752306 ^a^	67 (60.9%)	38 (34.5%)	5 (4.5%)	75 (77.3%)	21 (21.6%)	1 (1.0%)	<0.01*
*DAT1*	rs6347	81 (73.6%)	28 (25.5%)	1 (0.9%)	78 (80.4%)	19 (19.6%)	0 (0%)	0.21
	rs2975292	87 (79.1%)	22 (20.0%)	1 (0.9%)	70 (72.2%)	27 (27.8%)	0 (0.0%)	0.33
	rs37022	30 (27.3%)	55 (50.0%)	25 (22.7%)	16 (16.5%)	63 (64.9%)	18 (18.6%)	0.47
	rs40358	46 (41.8%)	48 (43.6%)	16 (14.3%)	37 (38.1%)	50 (51.5%)	10 (10.3%)	0.95
	rs10040882	87 (79.1%)	22 (20.0%)	1 (0.9%)	73 (75.3%)	24 (24.7%)	0 (0.0%)	0.63
	rs464049	50 (45.5%)	44 (40.0%)	16 (14.5%)	43 (44.3%)	47 (48.5%)	7 (7.2%)	0.5

^a^ The significant difference in this polymorphism (rs752306) between the case and control groups may have resulted from the limited number of participants.

* *p*<0.05

** *p*<0.1

### Lead exposure

There were challenges involved in obtaining blood during participant recruitment, especially for school-aged ADHD participants, and the difference in BLLs between the case and control groups was not significant. Therefore, we discontinued the blood collection after we had recruited 146 subjects (44 cases and 102 controls). The BLLs of these 146 subjects ranged from 0.44–4.71 μg/dL (Table 3), and lead was detected in 100% of the participants because of the low LOD at 0.001 μg/dL. The mean (± SD) BLLs in the control and ADHD groups were 1.73±0.77 μg/dL and 1.57±0.73 μg/dL, respectively. There was no significant difference in BLL between children with and without ADHD (*p* = 0.15). There was a small difference (<10%, data not shown) in the association between NP exposure and ADHD after adjusting for BLLs compared to the unadjusted association.

**Table 3:** Distribution of BLLs in control and ADHD participants (N = 146).

Substance (units)	Category	Sample Number	Detected (N)	Rate (%)	Mean±SD^a^	GM ^b^	Min	Percentile					Max	*p*-value^c^
								25%	50%	75%	90%	95%		
Lead (μg/dL)	Controls	102	102	100%	1.73±0.77	1.56	0.44	1.10	1.69	2.09	2.69	3.14	4.71	0.15
	ADHD	44	44	100%	1.57±0.73	1.43	0.64	0.97	1.44	1.98	2.52	3.46	3.81	
	Total	146	146	100%	1.68±0.76	1.52	0.44	1.07	1.63	2.05	2.62	3.19	4.71	

^a^ standard deviation

^b^ geometric mean

^c^ 2-sided Mann-Whitney U test.

### Urinary NP levels and association with ADHD

Urinary NP was detected in 97.6% of the participants (Table 4). The concentrations of NP ranged from ND (not detectable, less than 1.6 μg/L) to 18.38 μg/L. After creatinine adjustment, the mean (± SD) concentrations of NP in the control and ADHD groups were 4.64±2.95 and 4.52±3.22 μg/g cr., respectively. We did not observe a statistically significant relationship between urinary NP levels and the presence of ADHD (*p* = 0.43). Moreover, logistic regression analyses, with females included and excluded, suggested that children with creatinine-adjusted NP concentrations higher than the median may not present with a greater risk of having ADHD compared to children with concentrations below the median (Table 5). The odds ratios were essentially the same after adjusting for maternal education level and maternal alcohol exposure.

**Table 4:** Distribution of urinary NP (creatinine unadjusted and adjusted) in control and ADHD participants (N = 207).

Substance (units)	Category	Sample Number	Detected (N)	Rate (%)	Mean±SD^a^	GM ^b^	Min	Percentile					Max	*p*-value^c^
								25%	50%	75%	90%	95%		
NP (μg/L)	Controls	110	106	96.4%	4.23±2.50	3.65	ND	2.72	3.76	5.45	6.88	7.66	18.38	0.30
	ADHD	97	96	99.0%	3.99±2.16	3.51	ND	2.54	3.30	4.55	7.46	9.39	10.63	
	Total	207	202	97.6%	4.12±2.34	3.58	ND	2.65	3.52	5.02	7.00	8.63	18.38	
NP (μg/g cr.)	Controls	110	106	96.4%	4.64±2.95	3.79	0.34	2.77	3.92	5.86	9.05	10.35	16.83	0.43
	ADHD	97	96	99.0%	4.52±3.22	3.66	1.03	2.44	3.48	6.15	8.67	11.41	20.89	
	Total	207	202	97.6%	4.58±3.07	3.73	0.34	2.55	3.78	5.87	8.82	10.76	20.89	

^a^ standard deviation

^b^ geometric mean

^c^ 2-sided Mann-Whitney U test; ND: NP LOD = 1.6 μg/L, measurements below the LOD were assigned a value corresponding to LOD/2.

**Table 5:** Odds ratios for ADHD according to the creatinine-adjusted NP concentration.

NP concentration		Crude OR	95% CI	*p*-value	Adjusted OR^a^	95% CI	*p*-value
(A)	<median	1			1		
	≥median	0.72	0.42–1.24	0.24	0.68	0.38–1.22	0.19
(B)	<median	1			1		
	≥median	0.75	0.39–1.46	0.40	0.79	0.39–1.59	0.50

(A): all participants, N = 207; (B): boys, N = 144.

^a^ Adjusted covariates: maternal education levels and maternal drinking during pregnancy.

We detected a significant association between maternal drinking during pregnancy and ADHD (*p*<0.01), with few positive maternal drinking subjects reported in the control group. After exclusion, the limited number of participants with in utero alcohol exposure did not affect the adjusted odds ratio in the logistic regression analysis.

## Discussion

In this study, ADHD was significantly male predominant (male to female ratio: 5:1 in the ADHD group and 1.3:1 in the control group, *p*<0.01); ADHD was approximately three-fold more likely to be diagnosed in boys than in girls [56]. Our finding was in accord with previous studies [56, 57]. Biederman et al. suggested that girls with ADHD were less likely than boys to develop disruptive behavior disorder, a comorbid of ADHD, which may result in referral to specialists. The observation suggested that cases of girls with ADHD may be under-reported, which requires further attention [58, 59]. Because of the limited number of females with ADHD in this study (N = 16), we examined different analytical approaches to evaluate gender-related effects. A sensitivity analysis was performed by including and excluding females. The mean urinary NP levels in ADHD (N = 83) and non-ADHD (N = 61) boys in this study were 4.60±3.34 and 4.49±2.71 μg/g cr., respectively (*p* = 0.67, data not shown). Higher urinary NP levels did not increase the odds ratios between NP and ADHD when females were either included or excluded (Table 5). These data suggested that gender may not modify the relationship between urinary NP concentration and ADHD.

Several lines of evidence revealed that children with a family history of ADHD were more likely to be diagnosed with ADHD [32, 39, 49]. Our study found family history of nervous system diseases, including ADHD, was associated with higher risk for ADHD. Although the etiologies of various nervous system diseases are still unclear and may be different, there might be deficits in the neuroprotection function against assorted risk factors for the probands, and these conditions are consequently transmitted within families.

We found that high maternal and paternal educational attainment was associated with a decreased susceptibility for ADHD. This finding is in agreement with previous results indicating that low parental education levels are an important adverse risk factor for ADHD [32, 49, 60]. In this study, a statistically significant association was observed between maternal drinking and ADHD. Our finding is consistent with several studies that have linked prenatal alcohol exposure to ADHD [61–63]. These studies support the inclusion of alcohol as a risk factor for ADHD.

### Lead exposure of children in Taiwan

Lead exposure alters the dopamine system, which is relevant to the pathogenesis of ADHD [64, 65]. Low-level lead exposure has been associated with a clinical diagnosis of ADHD in several recent studies, even at concentrations much lower than the previous action level of 10 μg/dL (currently lowered to 5 μg/dL) [31, 34, 35, 66]. No significant difference in BLLs between the control and case groups was observed (1.73±0.77 μg/dL vs. 1.57±0.73 μg/dL, *p* = 0.15). The BLLs in this study were lower than those in the other two studies in Taiwan, which reported measurements of 5.50±1.86 μg/dL for 934 primary school children in 2002 and a range from 1.97 to 2.49 μg/dL for children 5–9 years of age in 2012 [67, 68]. Leaded gasoline has been banned in Taiwan since 2000. Meanwhile, 97.1% of the participants in the present study live in the greater Taipei area, which has few industrial emissions and tighter controls. These observations may explain the lower BLL measurements in this study compared to those in previous studies that were performed in 2002 and 2012.

### NP levels and detection rate compared to other studies

A previous study in this lab assessed the NP exposure of pubertal students aged 10.9–13.6 years and reported a geometric mean (GM) urinary NP level of 1.27 μg/g cr.; 30% of the urine samples were positive for NP [14]. In this current study of children aged 4–15 years, the urinary NP concentration GM was 3.73 μg/g cr. (Table 4), and the detection frequency was 97.6%. The NP levels in our samples were three-fold higher than those in pubertal students in Taiwan. Compared to studies from other nations, our measurements were approximately tenfold higher than reported levels in American adults (50% detection rate) and much higher than the readings in Belgium [69, 70]. NP was not detected in the urine samples of 131 subjects in a general Belgian population (LOD, 0.23 μg/L) [71]. However, the NP levels in the participants in our study (GM, 3.73 μg/g cr.) were lower than those measured in China, in which a GM of 15.92 μg/g cr. (100% detected) was reported for 287 children and students aged 3 to 24 years [72].

The differences in NP levels between our study and other reference values reported in the Belgian and American data may be caused by several factors. First, NPnEO, which are biodegraded to NP, are massively produced and widely used in Taiwan [73–75]. Second, NP is not effectively removed due to insufficient wastewater treatment in Taiwan [76, 77]. Third, detectable NP migrates into food from various plastic food containers and wrappings, which are widely used in Taiwanese daily activities [2, 78]. The relatively high levels of NP exposure and the high detection frequency in this study and in research performed in China indicate that children in Asian nations are consistently exposed to high levels of NP, which represents a significant concern.

### Association between NP exposure and the presence of ADHD

We report an absence of association between urinary NP concentrations and ADHD and no increased odds of ADHD for children with higher NP levels. The regression analyses, with and without excluding females, suggested that higher NP exposure may not promote the risk of having ADHD. Furthermore, no dose-response effects were observed.

According to Muller’s pharmacokinetic study, a bioavailability of 20% and a half-life of 2–3 h in blood were reported for NP. Moreover, only 10% of the applied dose is excreted in the urine [1]. Assuming that the volume of daily urine excretion is between 1000 and 1500 mL, the average daily body exposure of NP was estimated to be ~1.65–2.48 μg/day/Kg body weight (b.w.) for schoolchildren in Taiwan (data not shown). The daily NP exposure was calculated as follows:

Estimated daily exposure of NP (μg/day/Kg b.w.) = urinary NP levels (μg /L) x urine excretion per day (L/day) /10% NP exposure excreted through urine /body weight of participants (Kg)

The estimated daily exposure to NP in this study was lower than the tolerable daily intake (TDI) of 5 μg/day/Kg b.w. proposed by the Danish Institute of Food Safety and Toxicology [79]. Moreover, in Masuo’s animal study, young mice with a mean body weight of ~10 grams received an intracisternal injection of 87 nmol, which corresponds to 1.91 mg/Kg b.w. of NP exposure (NP has a molecular weight of 220 g/mole), approximately three orders of magnitude higher than the daily NP exposure in our study. An uncertainty factor of 1,000 typically was applied when deriving a no observed adverse effect level from experiments in laboratory animals to a reference dose for children [80]. However, for an intracisternal injection animal study, the adverse effects of a dose applied to the brain will be much more severe than those after oral intake. Thus, the daily NP exposures of children in our study were much lower compared to the intra-brain dose in mice that triggered spontaneous motor activity. This relatively low-dose exposure probably explains why our data do not support the hypothesis that exposure to endocrine-disrupting NP increases the risk of ADHD.

### Strengths and limitations

This study has a number of strengths. To the best of our knowledge, this is the first published study related to the association between pediatric urinary NP concentrations and ADHD in Taiwan. Moreover, the SNAP-IV questionnaire, a tool with acceptable reliability, was used to screen children with suspected ADHD. The diagnosis of ADHD was confirmed through an extensive evaluation based on the DSM-IV-TR clinical diagnosis performed by pediatricians or psychiatrists. Chances of misclassification between ADHD and non-ADHD were therefore minimal. In addition, we examined or adjusted for various important factors, including dopamine-related gene polymorphisms, exposure to lead, several socioeconomic indicators and maternal lifestyle factors.

However, this study also has several limitations. The limited sample size was related to the challenge of recruiting school-age children, especially those with ADHD, to participate in the study. The limited participation rate may be another limitation. Taipei City Hospital is a metropolitan hospital that serves the local population and accepts referrals from all the administrative districts in the city. In addition, 97.1% of the participants in the present study live in this metropolitan area. The cases and controls were recruited from the general pediatric clinics, not specific ADHD clinics, at the same hospital. Because attendance at Taipei City Hospital and the chance of being selected for the study were not dependent on NP levels, the chance of selection bias was probably low [81]. In addition, there was the potential for recall bias in our study, particularly regarding prenatal tobacco and alcohol exposure and family history of nervous diseases. The recall bias was minimized by (1) objectively measuring the variables of interest (urinary NP levels, BLLs and gene polymorphisms) and by (2) asking for information that did not depend heavily on memory or subjective interpretation, such as parental education levels and maternal age at birth.

## Conclusion

This case-control study found that NP exposure, at the estimated dosage of ~1.65–2.48 μg/day/kg b.w., was not a risk factor for ADHD in school-age children in Taiwan after adjusting for confounding factors. No differences were observed in BLLs between children with and without ADHD. More studies are warranted to further confirm the lack of a relationship between NP exposure and ADHD in children.
